# Artificial Intelligence in the Diagnosis of Hepatocellular Carcinoma: A Systematic Review

**DOI:** 10.3390/jcm11216368

**Published:** 2022-10-28

**Authors:** Alessandro Martinino, Mohammad Aloulou, Surobhi Chatterjee, Juan Pablo Scarano Pereira, Saurabh Singhal, Tapan Patel, Thomas Paul-Emile Kirchgesner, Salvatore Agnes, Salvatore Annunziata, Giorgio Treglia, Francesco Giovinazzo

**Affiliations:** 1 Department of Surgery, University of Illinois Chicago, Chicago, IL 60607, USA; 2 Faculty of Medicine, University of Aleppo, Aleppo 12212, Syria; 3 Department of Internal Medicine, King George’s Medical University, Lucknow 226003, Uttar Pradesh, India; 4 Faculty of Medicine, Universidad Complutense de Madrid, 28040 Madrid, Spain; 5 Department of HPB Surgery and Liver Transplantation, BLK-MAX Superspeciality Hospital, New Delhi 110005, Delhi, India; 6 Department of Surgery, Baroda Medical College and SSG Hospital, Vadodara 390001, Gujarat, India; 7 Département of Radiology and Medical Imaging, Cliniques Universitaires Saint-Luc, Institut de Recherche Expérimentale et Clinique, Université Catholique de Louvain, 1348 Brussels, Belgium; 8 General Surgery and Liver Transplantation Unit, Fondazione Policlinico Universitario Agostino Gemelli IRCCS, 00168 Rome, Italy; 9 Unit of Nuclear Medicine, Department of Radiology, Radiotherapy and Hematology, Fondazione Policlinico Universitario A. Gemelli IRCCS, 00168 Rome, Italy; 10 Imaging Institute of Southern Switzerland, Ente Ospedaliero Cantonale, 6500 Bellinzona, Switzerland; 11 Faculty of Biomedical Sciences, Università della Svizzera Italiana, 6900 Lugano, Switzerland; 12 Faculty of Biology and Medicine, University of Lausanne, 1015 Lausanne, Switzerland

**Keywords:** artificial intelligence, deep learning, diagnosis, hepatocellular carcinoma, HCC, machine learning

## Abstract

Hepatocellular carcinoma ranks fifth amongst the most common malignancies and is the third most common cause of cancer-related death globally. Artificial Intelligence is a rapidly growing field of interest. Following the PRISMA reporting guidelines, we conducted a systematic review to retrieve articles reporting the application of AI in HCC detection and characterization. A total of 27 articles were included and analyzed with our composite score for the evaluation of the quality of the publications. The contingency table reported a statistically significant constant improvement over the years of the total quality score (*p* = 0.004). Different AI methods have been adopted in the included articles correlated with 19 articles studying CT (41.30%), 20 studying US (43.47%), and 7 studying MRI (15.21%). No article has discussed the use of artificial intelligence in PET and X-ray technology. Our systematic approach has shown that previous works in HCC detection and characterization have assessed the comparability of conventional interpretation with machine learning using US, CT, and MRI. The distribution of the imaging techniques in our analysis reflects the usefulness and evolution of medical imaging for the diagnosis of HCC. Moreover, our results highlight an imminent need for data sharing in collaborative data repositories to minimize unnecessary repetition and wastage of resources.

## 1. Introduction

Artificial intelligence (AI) is “a field of science and engineering concerned with the computational understanding of what is commonly called intelligent behavior, and with creating artefacts that exhibit such behavior” [1].

Alan Turing first described the use of computers for the simulation of critical thinking and intelligence in 1950. In 1956, John McCarthy coined the definition of AI, the all-encompassing term for computer programs replicating human intelligence. Machine learning is a subset of AI that trains on learning from previous experience and rectifies its functioning sequentially. Deep learning (DL) is a further subset of machine learning that utilizes multi-layered networks between the computing units termed “neurons” that process and validate large training datasets between input and output units, and it leads to meaningful predictions in multiple spheres of medical research (diagnostic, therapeutic, prognostic, etc.) [2].

Hepatocellular carcinoma (HCC) ranks fifth amongst the most common malignancies and is the third most common cause of cancer-related death globally [3]. Though there have been several breakthroughs in the treatment and diagnostic capability, the prognosis of HCC remains dismal due to delayed diagnosis and limited treatment strategies. AI has far reaching potential in the sphere of (a) risk factor stratification, (b) characterization, and (c) improved prognostication in established cases [2]. HCC is a notorious cancer with multiple and overlapping risk factors with the spectrum of its evolving conditions, including NAFLD (Non-Alcoholic Fatty liver disease), NASH (Non-Alcoholic steatohepatitis), and subsequent cirrhosis. Several AI modalities have now been modelled to differentiate and predict the risk of incident HCC [2]. The next challenge lies in classifying indeterminate liver lesions requiring histopathological evidence. The use of computed tomography (CT) and magnetic resonance imaging (MRI) based on DL and radiomics and the success in differentiating between HCC and non-HCC liver nodules with high diagnostic accuracy serve as an essential impetus for creating universal standardized liver tumor segmentation techniques [4]. The following systematic review will expand on the current role of artificial intelligence in HCC detection and characterization, regardless of the instrumental technique.

## 2. Materials and Methods

Following the Preferred Reporting Items for Systematic Reviews and Meta-analyses (PRISMA) reporting guidelines, we conducted a systematic review. This review reports qualitative data, and because of inconsistent reporting of outcome measures and differences in populations and study design, we did not perform a meta-analysis.

### 2.1. Searches

PubMed, Scopus, and Cochrane were searched using a combination of the following key words: ((Artificial Intelligence) OR (Machine Learning)) AND ((Hepatocellular Carcinomas) OR (HCC) OR (Liver Cancer)) to retrieve articles reporting the application of AI in detecting or diagnosing HCC. Results were admitted from the time of inception up to and including 5 May 2022. The search terms were modified to fit each database (the terms and their adjustments are found in the Supplementary Materials File S1). Additionally, the reference list of included articles and relevant reviews was checked manually to identify other papers.

### 2.2. Inclusion and Exclusion Criteria

Only published articles reporting the application of AI in detecting or diagnosing HCC were included, excluding all the studies reporting the application of AI outside the Diagnosis of HCC, such as risk prediction, prognosis, or treatment. Only diagnoses based on CT, MRI, Ultrasound (US), 18F-FDG Positron emission tomography (PET), or X-ray were selected, while other methods like pathology reports or biomarkers were excluded. Reviews, letters, editorials, conference papers, preprints, commentaries, book chapters, or any article in languages other than English were excluded too.

### 2.3. Quality Assessment

Studies were assessed for quality based on three items:

- The number of images, estimating the risk of bias and overfitting: fewer than 50 (score 0), 50 to 100 (score 1), and more than 100 (score 2) [5]. This factor was considered the most frequently reported in articles. Where only the number of patients was reported, we considered at least one image per patient.
- The use of a completely independent cohort for validation: no cohort (score 0), the partition of the cohort between completely separated training and test set (score 1), external validation cohort (score 2).
- By 2011, the speed of graphics processing units had increased significantly, making it possible to train convolutional neural networks “without” the layer-by-layer pre-training. With the increased computing speed, deep learning had significant advantages in terms of efficiency and speed: no data (score 0), before 2011 (score 1), 2011 or after (score 2).

A simple quality score (QS), consisting of the sum of the 3 previously stated items, was calculated. A maximum possible score of 6 meant a high-quality study design of the article.

### 2.4. Study Selection & Data Extraction

Duplicates were removed using Endnote X9. Titles and/or abstracts of studies identified using our search criteria were screened independently by 2 authors (A.M. & M.A.) to identify all studies meeting our inclusion criteria. Any disagreement was resolved through discussion with a third reviewer (F.G.). Random included articles were used to generate an extraction sheet. Three authors (A.M., M.A., and J.P.S.P) reviewed the full texts for inclusion and data extraction. Any discrepancies were corrected by consensus. The following parameters were extracted from each article:

- PMID; First author; Year of publication; Country; Journal.
- The number of patients; Diagnostic method; AI method.
- Research question; Key findings.
- Quality score.

F.G. then reviewed all articles, rechecked data, and analyzed them using an Excel (R) sheet. Statistical calculations were performed with Jamovi (R) software version 2.0.0.0 [6,7].

## 3. Results

### 3.1. Searching Results

**Figure 1:**
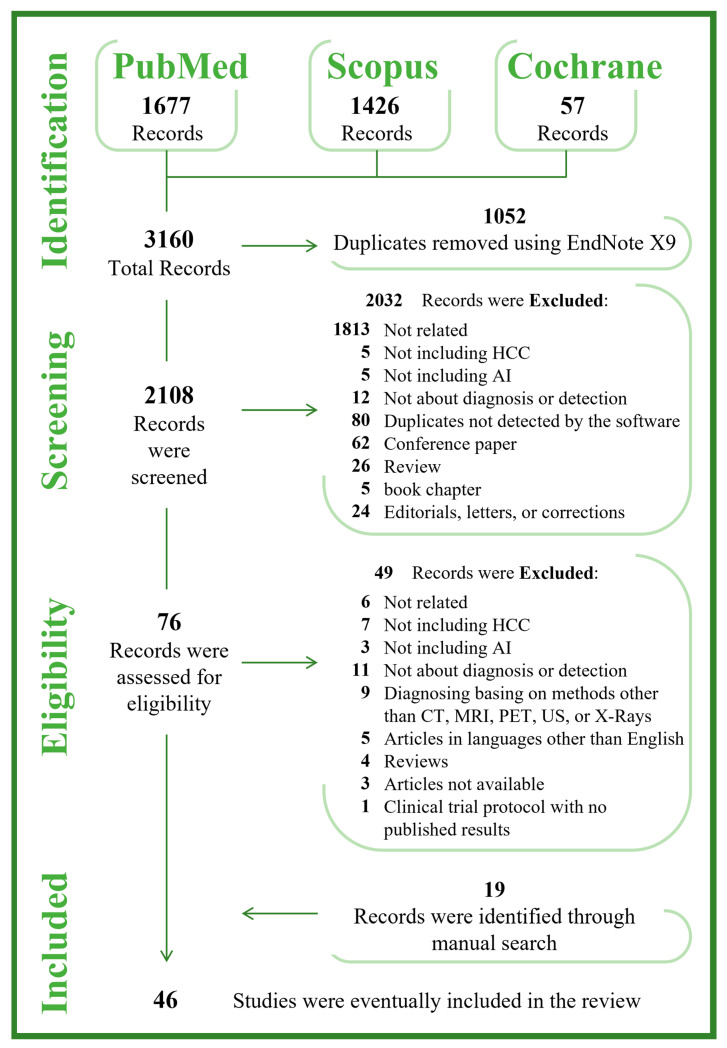
PRISMA Flow Diagram.

The study flow diagram is illustrated in Figure 1. Searches identified 3160 records: 1677 from PubMed, 1426 from Scopus, and 57 from Cochrane. A total of 1052 were duplicates and automatically excluded using EndNoteX9. A total of 2108 studies were evaluated by title/abstract screening against the eligibility criteria, and 2032 were excluded. Of these, 1813 were not related to the topic, 5 not including HCC, 5 not including AI, 12 not discussing diagnosis, 80 duplicates not detected by the software, 62 conference papers, 26 reviews, 5 book chapters, and 24 letters. Of the remaining 76 records potentially eligible, after the full-texts screening, 27 articles were included, and 49 were excluded because 6 were not related to the topic, 7 did not include HCC, 3 did not include AI, 11 did not discuss diagnosis, 9 diagnosed based on methods other than CT/PET/MRI/US/X-ray, 5 articles were in a language other than English, 4 were reviews, 3 articles were not available, and 1 was a clinical trial with no published data. After the manual search, 19 articles were further identified. Thus, a total of 46 cited articles were included in this review, published between 1998 and 2022 (Table 1).

### 3.2. Quality Assessments

The mean of the “Number of Images” score was 1.70, identifying 36 articles (78.3%) where at least 100 images were analyzed. (Table 2) The mean of the “Cohort for Validation” Score was 0.609. Indeed, an external validation cohort was used only in 2 articles (4.3%). (Table 3) The mean of the “Year of Publication” score was 1.87, documenting that most of the works (87.0%) included in this systematic review were published in 2011 or later. (Table 4) On average, the Total Quality Score was 4.17, with a median of 4.00 and SD of 1.04. (Table 5) The contingency table correlating the Total Score with the Year of Publication reports a statistically significant constant improvement over the years of the quality score (*p* = 0.004). (Table 6) A total of 3 articles (6.52%) scored a QS lower than 3, while 2 (4.34%) received the maximum score. Results from articles with a QS strictly lower than 3 are written in italics in Table 7.

### 3.3. Results

Different AI methods have been adopted in the included articles, such as CNN (Convolutional Neural Network), SVM (Support-Vector Machine), RF (Random Forest), KNN (K-Nearest Neighbor), PM-DL (pattern matching and deep learning), ANN (Artificial Neural Network), DNN (Deep Neural Network), CDNs (Convolutional Dense Networks), DLS (Deep Learning System), GLM (Generalized Linear Model), DWT (Discrete Wavelet Transform), LSTM (Long Short-Term Memory), NNE (Neural Network Ensemble), and LDA (Linear Discriminant Analysis). A total of 19 articles used CT (41.30%), 20 used US (43.47%), and 7 used MRI (15.21%) in their work. No article has discussed the use of artificial intelligence in PET and X-ray technology. Table 7 lists the total study population, diagnostic method, research question or purpose, AI method, key findings included in this systematic review to summarize how artificial intelligence is used today in diagnosing HCC. Moreover, when the information was available, we reported in Table 7 the background of the images studied, i.e., whether HCC on the cirrhotic or healthy liver and whether other cancerous and benign lesions studied were present.

**Table 7:** Characteristics of the studies included in the systematic review.

Study	Country/Region	Journal	Total Study Population	Diagnostic Method	Research Question/Purpose	AI Method	Key Findings
Ziegelmayer et al., 2022 [8]	Germany	*Investigative Radiology*	60 patients	CT	To compare the robustness of CNN features versus radiomics features to technical variations in image acquisition parameters.	CNN	CNN features were more stable.
Xu et al., 2022 [9]	China	*Computational and Mathematical Methods in Medicine*	211 patients (122 training set, 89 testing set)	CT	To establish an SVM based on radiomic features at non-contrast CT to train a discriminative model for HCC and ICCA at early stage.	SVM	The model may facilitate the differential diagnosis of HCC and ICCA in the future.
Turco et al., 2022 [10]	USA	*IEEE Transactions on Ultrasonics, Ferroelectrics, and Frequency control*	72 patients	US	Proposes an interpretable radiomics approach to differentiate between malignant and benign FLLs on CEUS.	Logistic regression, SVM, RF, and KNN	Aspects related to perfusion (peak time and wash-in time), the microvascular architecture (spatiotemporal coherence), and the spatial characteristics of contrast enhancement at wash-in (global kurtosis) and peak (GLCM Energy) are particularly relevant to aid FLLs diagnosis.
Sato et al., 2022 [11]	Japan	*Journal of Gastroenterology and Hepatology*	972 patients (864 training set, 108 testing set)	US	To analyse the diagnostic performance of deep multimodal representation model-based integration of tumour image, patient background, and blood biomarkers for the differentiation of liver tumours observed using B-mode US.	CNN	The integration of patient background information and blood biomarkers in addition to US images, multi-modal representation learning outperformed the CNN model that used US images alone.
Rela et al., 2022 [12]	India	*International Journal of Advanced Technology and Engineering Exploration*	68 patients (51 training set, 17 testing set)	CT	Different machine learning algorithms are used to classify the tumour as liver abscess and HCC.	SVM, KNN, Decision tree, Ensemble, and Naive Bayes	SVM classifier gives better performance compared to all other AI methods in the study.
Zheng et al., 2021 [13]	China	*Physics in Medicine and Biology*	120 patients (56 training set with 5376 images, 64 testing set with 6144 images)	MRI	To investigate the feasibility of automatic detection of small HCC (≤2 cm) based on PM-DL model.	CNN	The superior performance both in the validation cohort and external test cohort indicated the proposed PM-DL model may be feasible for automatic detection of small HCCs with high accuracy.
Yang et al., 2021 [14]	Taiwan	*PLoS One*	731 patients (394 training set with 10,130 images, 337 testing set with 22,116 images)	CT	To use a previously proposed mask region–based CNN for automatic abnormal liver density detection and segmentation based on HCC CT datasets from a radiological perspective.	CNN	The study revealed that this single deep learning model cannot replace the complex and subtle medical evaluations of radiologists, but it can reduce tedious labour.
Stollmayer et al., 2021 [15]	Hungary	*World Journal of Gastroenterology*	69 patients (training set with 186 images, testing set with 30 images)	MRI	To compare diagnostic efficiency of 2D and 3D-densely connected CNN (DenseNet) for FLLs on multi-sequence MRI.	CNN	Both 2D and 3D-DenseNets can differentiate FNH, HCC and MET with good accuracy when trained on hepatocyte-specific contrast-enhanced multi-sequence MRI volumes.
Kim et al., 2021 [16]	Korea	*European Radiology*	1320 patients (training set with 568 images, testing set with 589 images, tuning set with 193 images)	CT	To develop and evaluate a deep learning-based model capable of detecting primary hepatic malignancies in multiphase CT images of patients at high risk for HCC.	CNN	The proposed model exhibited an 84.8% of sensitivity with 4.80 false positives per CT scan in the test set.
Căleanu et al., 2021 [17]	Romania	*Sensors*	91 patients	US	To examine the application of CEUS for automated FLL diagnosis using DNN.	DNN	This deep learning-based method provides comparable or better results, for an increased number of FLL types.
Zhou et al., 2020 [18]	China	*Frontiers in Oncology*	435 patients (616 liver lesions; 462 training set, 154 testing set)	CT	To propose a framework based on hierarchical CNNs for automatic detection and classification FLLs in multi-phasic CT.	Hierarchical CNNs	Overall, this preliminary study demonstrates that the proposed multi-modality and multi-scale CNN structure can locate and classify FLLs accurately in a limited dataset and would help inexperienced physicians to reach a diagnosis in clinical practice.
Kim et al., 2020 [19]	South Korea	*Scientific Reports*	549 patients, and external validation data set (54 patients)	MRI	To develop a fully automated deep learning model to detect HCC using hepatobiliary phase MR images and evaluate its performance in detecting HCC on liver MRI compared to human readers	Fine-tuned CNN	The optimised CNN architecture achieved 94% sensitivity, 99% specificity, and 0.97 area under curve (AUC) for HCC cases in the test dataset and achieved 87% sensitivity and 93% specificity and an AUC of 0.90 for external validation datasets.
Huang et al., 2020 [20]	China	*IEEE journal of biomedical and health informatics*	Data set 1: 155 patients with FNH and 49 patients with atypical HCC Data set 2: 102 patients with FNH and only 36 patients with atypical HCC	US	To propose a novel liver tumour CAD approach extracting spatial-temporal semantics for atypical HCC.	SVM	The average accuracy reaches 94.40%, recall rate 94.76%, F1-score value 94.62%, specificity 93.62% and sensitivity 94.76%.
Shi et al., 2020 [21]	NA	*Abdominal Radiology*	342 patients with 449 untreated lesions (194 HCC group; 255 non-HCC group)	CT	To evaluate whether a three-phase dynamic contrast-enhanced CT protocol, when combined with a deep learning model, has similar accuracy in differentiating HCC from other FLLs) compared with a four-phase protocol.	CDNs	When combined with a CDN, a three-phase CT protocol without pre-contrast showed similar diagnostic accuracy as a four-phase protocol in differentiating HCC from other FLLs, suggesting that the multiphase CT protocol for HCC diagnosis might be optimised by removing the pre-contrast phase to reduce radiation dose.
Zhen et al., 2020 [22]	China	*Frontiers in Oncology*	1210 patients (31,608 images), and external validated cohort of 201 patients (6816 images)	MRI	To develop a DLS to classify liver tumours.	CNN	DLS that integrated these models could be used as an accurate and timesaving assisted-diagnostic strategy for liver tumours in clinical settings, even in the absence of contrast agents. DLS therefore has the potential to avoid contrast-related side effects and reduce economic costs associated with current standard MRI inspection practices for liver tumour patients.
Krishan et al., 2020 [23]	India	*Proceedings of the Institution of Mechanical Engineers, Part H: Journal of Engineering in Medicine*	794 normal liver images and 844 abnormal liver types (483 MET, 361 HCC)	CT	To detect the presence of a tumour region in the liver and classify the different stages of the tumour from CT images.	R-part decision tree, AdaBoost, RF, k-SVM, GLM, and NN. A multi-level ensemble model is also developed.	The accuracy achieved for different classifiers varies between 98.39% and 100% for tumour identification and between 76.38% and 87.01% for tumour classification. The multi-level ensemble model achieved high accuracy in both the detection and classification of different tumours.
Brehar et al., 2020 [24]	Romania	*Sensors*	268 patients	US	To compare deep-learning and conventional machine-learning methods for the automatic recognition of the HCC areas from US images	CNNs	The achieved results show that the deep-learning approach overcomes classical machine-learning solutions, by providing a higher classification performance.
Hamm et al., 2019 [25]	NA	*European radiology*	296 patients; 334 imaging studies; 494 hepatic lesions divided into training (434) and test sets (60)	MRI	To develop and validate a proof-of-concept CNN–based DLS that classifies common hepatic lesions on multi-phasic MRI.	CNN	This preliminary deep learning study demonstrated feasibility for classifying lesions with typical imaging features from six common hepatic lesion types.
Das et al., 2019 [26]	India	*Pattern Recognition and Image Analysis*	123 real-time images (63 HCC, and 60 metastasis carcinoma)	CT	To present an automatic approach that integrates the adaptive thresholding and spatial fuzzy clustering approach for detection of cancer region in CT scan images of liver.	Multilayer perceptron and C4.5 decision tree classifiers	This result proves that the spatial fuzzy c-means-based segmentation with C4.5 decision tree classifier is an effective approach for automatic recognition of the liver cancer.
Trivizakis et al., 2019 [27]	Greece	*IEEE Journal of Biomedical and Health Informatics*	130 images for the training and validation of the network	MRI	Propose and evaluate a novel 3D CNN designed for tissue classification in medical imaging and applied for discriminating between primary and metastatic liver tumours from diffusion weighted MRI data.	3D CNN	The proposed 3D CNN architecture can bring significant benefit in diffusion weighted MRI liver discrimination and potentially, in numerous other tissue classification problems based on tomographic data, especially in size-limited, disease-specific clinical datasets.
Kutlu et al., 2019 [28]	Turkey	*Sensors*	56 images benign and 56 malignant liver tumours	CT	A new liver and brain tumour classification method is proposed by using the power of CNN in feature extraction, the power of DWT in signal processing, and the power of LSTM in signal classification.	CNN in feature extraction, DWT in signal processing, and LSTM in signal classification	The proposed method has a satisfactory accuracy rate at the liver tumour and brain tumour classifying.
Nayak et al., 2019 [29]	India	*International Journal of Computer Assisted Radiology and Surgery*	40 patients (healthy 14, cirrhosis 12, and cirrhosis with HCC 14)	CT	To proposes a CAD system for detecting cirrhosis and HCC in a very efficient and less time-consuming approach.	SVM	The proposed CAD system showed promising results and can be used as effective screening tool in medical image analysis.
Schmauch et al., 2019 [30]	France	*Diagnostic and Interventional Imaging*	544 patients (367 training set, 177 test set)	US	To create an algorithm that simultaneously detects and characterises (benign vs. malignant) FLL using deep learning.	ANN	This method could prove to be highly relevant for medical imaging once validated on a larger independent cohort.
Jansen et al., 2019 [31]	The Netherlands	*PLoS ONE*	95 patients (213 images)	MRI	Additional MR sequences and risk factors are used for automatic classification.	Randomised trees classifier	The proposed classification can differentiate five common types of lesions and is a step forward to a clinically useful aid.
Das et al., 2019 [32]	India	*Cognitive Systems Research*	75 patients (225 images)	CT	To introduce a new automated technique based on watershed–Gaussian segmentation approach.	DNN	The developed system is ready to be tested with huge database and can aid the radiologist in detecting the liver cancer using CT images.
Mokrane et al., 2019 [33]	France	*European Radiology*	178 patients (142 training set, 36 validations set)	CT	To enhance clinician’s decision-making by diagnosing HCC in cirrhotic patients with indeterminate liver nodules using quantitative imaging features extracted from triphasic CT scans.	KNN, SVM, and RF	A proof of concept that machine-learning-based radiomics signature using change in quantitative CT features across the arterial and portal venous phases can allow a non-invasive accurate diagnosis of HCCs in cirrhotic patients with indeterminate nodules.
Lakshmipriya et al., 2019 [34]	India	*Journal of Biomedical and Health Informatics*	634 images (440 images training set, 194 images validation set)	CT	An ensemble FCNet classifier is proposed to classify hepatic lesions from the deep features extracted using GoogleNetLReLU transfer learning approach.	CNN	Results demonstrate the efficacy of the proposed classifier design in achieving better classification accuracy.
Acharya et al., 2018 [35]	Malaysia	*Computers in biology and medicine*	101 patients with 463 images	US	This study initiates a CAD system to aid radiologists in an objective and more reliable interpretation of ultrasound images of liver lesions.	Radon transform and bi-directional empirical mode decomposition to extract features from the focal liver lesions.	The accuracy, sensitivity, and specificity of lesion classification were 92.95%, 90.80%, and 97.44%, respectively.
Ta et al., 2018 [36]	USA	*Radiology*	106 images (54 malignant, 51 benign, and one indeterminate FLL)	US	To assess the performance of CAD systems and to determine the dominant US features when classifying benign versus malignant FLLs by using contrast material–enhanced US cine clips.	ANN and SVM	CAD systems classified benign and malignant FLLs with an accuracy like that of an expert reader. CAD improved the accuracy of both readers. Time-based features of TIC were more discriminating than intensity-based features.
Bharti et al., 2018 [37]	India	*Ultrasonic Imaging*	94 patients (189 images)	US	To deal with this difficult visualisation problem, a method has been developed for classifying four liver stages, that is, normal, chronic, cirrhosis, and HCC evolved over cirrhosis.	KNN, SVM, rotation forest, CNNs	The experimental results strongly suggest that the proposed ensemble classifier model is beneficial for differentiating liver stages based on US images.
Yasaka et al., 2018 [38]	Japan	*Radiology*	560 patients (460 patients training set with 55,536 images, 100 patients validation set with 100 images)	CT	To investigate diagnostic performance by using a deep learning method with a CNN for the differentiation of liver masses at dynamic contrast agent-enhanced CT.	CNN	Deep learning with CNN showed high diagnostic performance in differentiation of liver masses at dynamic CT.
Hassan et al., 2017 [39]	Egypt	*Arabian Journal for Science and Engineering*	110 patients (110 images)	US	A new classification framework is introduced for diagnosing focal liver diseases based on deep learning architecture.	Stacked Sparse Autoencoder	Our proposed method presented overall accuracy of 97.2% compared with multi-SVM, KNN, and Naïve Bayes.
Guo et al., 2017 [40]	China	*Clinical Hemorheology and Microcirculation*	93 patients	US	To propose a novel two-stage multi-view learning framework for the CEUS based CAD for liver tumours, which adopted only three typical CEUS images selected from the arterial phase, portal venous phase and late phase.	Deep canonical correlation analysis and multiple kernel learning	The experimental results indicate that the proposed achieves best performance for discriminating benign liver tumours from malignant liver cancers.
Kondo et al., 2017 [41]	Japan	*Transactions on Medical Imaging*	98 patients	US	To propose an automatic classification method based on machine learning in CEUS of FLLs using the contrast agent Sonazoid.	SVM	The results indicated that combining the features from the arterial, portal, and post-vascular phases was important for classification methods based on machine learning for Sonazoid CEUS.
Gatos et al., 2015 [42]	NA	*Medical physics*	52 patients; (30 benign and 22 malignant)	US	Detect and classify FLLs from CEUS imaging by means of an automated quantification algorithm.	SVMs	The proposed quantification system that employs FLLs detection and classification algorithms may be of value to physicians as a second opinion tool for avoiding unnecessary invasive procedures.
Virmani et al., 2014 [43]	India	*Journal of Digital Imaging*	108 images	US	An NNE-based CAD system to assist radiologists in differential diagnosis between FLLs.	NNE	The promising results obtained by the proposed system indicate its usefulness to assist radiologists in differential diagnosis of FLLs.
Wu et al., 2014 [44]	China	*Optik*	22 patients	US	To propose a diagnostic system for liver disease classification based on CEUS imaging.	DNN	Quantitative comparisons demonstrate that the proposed method outperforms the compared classification methods in accuracy, sensitivity, and specificity
Virmani et al., 2013 [45]	India	*Defence Science Journal*	108 images comprising of 21 NOR images, 12 Cyst, 15 HEM, 28 HCC, and 32 MET	US	To investigate the contribution made by texture of regions inside and outside of the lesions in FLLs.	SVM	The proposed PCA-SVM based CAD system yielded classification accuracy of 87.2% with the individual class accuracy of 85%, 96%, 90%, 87.5%, and 82.2% for NOR, Cyst, HEM, HCC, and MET cases, respectively. The accuracy for typical, atypical, small HCC and large HCC cases is 87.5%, 86.8%, 88.8%, and 87%, respectively.
Streba et al., 2012 [46]	Romania	*World Journal of Gastroenterology*	224 patients	US	To study the role of time-intensity curve analysis parameters in a complex system of neural networks designed to classify liver tumours.	ANN	Neural network analysis of CEUS-obtained time-intensity curves seem a promising field of development for future techniques, providing fast and reliable diagnostic aid for the clinician.
Mittal et al., 2011 [47]	India	*Computerized Medical Imaging and Graphics*	88 patients with 111 images comprising 16 normal liver, 17 Cyst, 15 HCC, 18 HEM and 45 MET	US	It proposes a CAD system to assist radiologists in identifying focal liver lesions in B-mode ultrasound images.	Two step neural network classifier	The classifier has given correct diagnosis of 90.3% (308/340) in the tested segmented regions-of-interest from typical cases and 77.5% (124/160) in tested segmented regions-of-interest from atypical cases.
Sugimoto et al., 2010 [48]	Japan	*World Journal of Radiology*	137 patients (74 HCCs, 33 liver metastases and 30 liver hemangiomas)	US	To introduce CAD aimed at differential Diagnosis of FLLs by use of CEUS.	ANNs	The classification accuracies were 84.8% for metastasis, 93.3% for hemangioma, and 98.6% for all HCCs. In addition, the classification accuracies for histologic differentiation types of HCCs were 65.2% for w-HCC, 41.7% for m-HCC, and 80.0% for *p*-HCC.
Shiraishi et al., 2008 [49]	Japan	*Medical Physics*	97 patients, (103 images; 26 metastases, 16 hemangiomas, and 61 HCCs)	US	To develop a CAD scheme for classifying focal liver lesions as liver metastasis, hemangioma, and three histologic differentiation types of HCC, by use of microflow imaging of CEUS.	ANNs	The classification accuracies for the 103 FLLs were 88.5% for metastasis, 93.8% for hemangioma, and 86.9% for all HCCs. In addition, the classification accuracies for histologic differentiation types of HCCs were 79.2% for w-HCC, 50.0% for m-HCC, and 77.8% for *p*-HCC.
Stoitsis et al., 2006 [50]	Greece	*Nuclear Instruments and Methods in Physics Research*	147 images (normal liver 76, hepatic cyst 19, hemangioma 28, HCC 24)	CT	To classify of four types of hepatic tissue: normal liver, hepatic cyst, hemangioma, and hepatocellular carcinoma, from CT images.	Combined use of texture features and classifiers	The achieved classification performance was 100%, 93.75%, and 90.63% in the training, validation, and testing set, respectively.
Matake et al., 2006 [51]	NA	*Academic radiology*	120 patients	CT	To apply an ANN for differential diagnosis of certain hepatic masses on CT images and evaluate the effect of ANN output on radiologist diagnostic performance.	ANN	The ANN can provide useful output as a second opinion to improve radiologist diagnostic performance in the differential diagnosis of hepatic masses seen on contrast-enhanced CT.
Gletsos et al., 2003 [52]	Greece	*IEEE transactions on information technology in biomedicine*	147 patients	CT	To present a CAD system for the classification of hepatic lesions from CT images.	Neural-Network Classifier	The suitability of co-occurrence texture features, the superiority of GAs for feature selection, compared to sequential search methods, and the high performance achieved by the NN classifiers in the testing images set have been demonstrated.
Chen et al., 1998 [53]	Taiwan	*IEEE Transactions on Biomedical Engineering*	30 patients	CT	To present a CT liver image diagnostic classification system which will automatically find, extract the CT liver boundary, and further classify liver diseases.	Modified probabilistic NN	The proposed system was evaluated by 30 liver cases and shown to be efficient and very effective.

AI: Artificial Intelligence; CT: Computerized Tomography; CNN: Convolutional Neural Network; SVM: Support-Vector Machine; HCC: Hepatocellular Carcinoma; ICCA: Intrahepatic Cholangiocarcinoma; CEUS: Contrast Enhanced Ultrasound; FLL: Focal Liver Lesion; RF: Random Forest; KNN: K-Nearest Neighbor; US: Ultrasound; MRI: Magnetic Resonance Imaging; PM-DL: pattern matching and deep learning; 2D: Two-Dimensional; 3D: Three-Dimensional; FNH: Focal Nodular Hyperplasia; MET: Metastatic; ANN: Artificial Neural Network; DNN: Deep Neural Network; CAD: Computer-Aided Diagnosis; NA: Not Applicable; CDNs: Convolutional Dense Network; DLS: Deep Learning System; GLM: Generalized Linear Model; NN: Neural Network; DWT: Discrete Wavelet Transform; LSTM: Long Short-Term Memory; NNE: Neural Network Ensemble; LDA: Linear Discriminant Analysis.

## 4. Discussion

Artificial Intelligence is a rapidly growing field of interest. It has immense potential to be the standard of care in resource-limited settings where there is lesser availability of expert care and a heavy burden of cancer volume load. However, the use of AI and ML-based algorithms are limited in current practice owing to their limited generalizability. ML algorithms require large training sets of data, processing using GPUs and functions on the GIGO (Garbage in Garbage out) principle, which means that the output is as robust as the input obtained. However, robustness and standardization of large datasets, including follow-up evaluation and patient quality of care, is extremely cumbersome and difficult. The incongruity between modelled datasets versus real-world data is a fundamental challenge that must be overcome in the future [2].

We have grouped systemically the articles using artificial intelligence in HCC detection and characterization in a unique table, helping to plan further research projects. Indeed, for each article, we extracted the scope, the AI method used, and the key findings related to that AI approach, with the idea of having an index of all projects carried out to date. The significant heterogeneity of the studies reflected the difficulty of extrapolating several variables related to the different radiological techniques and pulling them together (e.g., gold standard used for the diagnosis of HCC, patient features, radiologist’s opinion, dose and type of contrast agent, and follow-up imaging).

In this work, 27 articles were analyzed with our composite score for the evaluation of the quality of the publications, with an overall score at 4.17/6. The “Cohort for Validation” score was the lowest, indeed, an external validation cohort was used only in 2 articles. This phenomenon, although explained by the difficulty of collecting data, limits the generalizability of the conclusions. We observe a statistically significant constant improvement over the years on our composite criterion combining the number of images and the presence of a validation cohort. (*p* = 0.004) This improvement is probably due to the publication of guidelines, dedicated checklists to ensure proper methodology, and technological improvement in the field of AI.

Our results highlight an imminent need for data sharing in collaborative data repositories to minimize unnecessary repetition and wastage of resources. In addition, universal standardized data sharing protocols for sharing datasets from clinical trials are essential to help make the available data robust and fill in the missing data. One such example is the creation of the Human Brain Project and project EBRAINS by the European Union to handle data related to brain research and its broader usage in the development of AI networks [54]. To help make the datasets uniformly accessible and usable, it is also imperative to diversify the data. Most of the work on AI-based algorithms was done on small-scale datasets due to economic and logistic constraints in high-income developed countries with limited to no data from lower middle- and low-income countries, which puts their credibility in ambiguity. Significant work needs to be done to increase the transparency and understanding of AI algorithms so that healthcare professionals gain confidence in using them in clinical settings.

Our systematic approach has shown that previous works in HCC detection and characterization have assessed the comparability of conventional interpretation with machine learning using US, CT, and MRI. The distribution of the imaging techniques in our analysis reflects the usefulness and evolution of medical imaging for the diagnosis of HCC. Ultrasound and CT are overrepresented in our analysis as both are easily available imaging techniques that have long proven their usefulness in the diagnosis of HCC. More recent and limited access to MRI may explain its absence before and low representation since 2019 in our analysis. On the opposite side, no study investigated X-ray or PET techniques. Indeed, even if X-ray has an interesting role in interventional therapeutic procedures, this technique has not been used for diagnostic purposes in this field. Moreover, unlike the other branches of medicine, such as neurology [55], head and neck cancer [56], or lung imaging [57,58,59], artificial intelligence in PET technology has not yet been studied and tested in HCC diagnosis. As PET, in combination with CT scan, is already used in other cancer to define undetermined lesions with high sensitivity and precision, AI and PET technology in HCC have not been explored yet. The most straightforward explanation can be found in the difficulty in analyzing structural and morphological characteristics, the hepatocellular cancer lesions having a variable degree of avidity for the PET tracers such as 18F-FDG. Indeed, the liver is unique in its capacity to maintain glucose homeostasis, thus leading to low 18F-FDG uptakes in low-grade (i.e., relatively metabolically less active) tumors [60]. It has been reported that only up to two-thirds of the tumors are 18F-FDG avid, although higher standardized uptake values (SUV) indicate a more malignant tumor [61,62]. Using other tracers such as 18F-Choline and 11C-Acetate may be a promising approach to increase the accuracy of results and openness to new AI technologies in combination with PET in diagnosing HCC [63,64].

In the future, DL algorithms combining clinical, radiological, pathological, and molecular information can help identify and better prognosticate patients. In addition, algorithms trained on post-chemotherapy patients could help in the early identification of their response and the time to switch between other therapeutic options. This will enable earlier identification of patients with poor treatment response and pre-emptive therapy adjustment based on molecular signature and imaging [2,4]. Anyhow, conducting high quality AI studies with large sets of data remains a real challenge whatever the medical imaging technique. Supervised and moreover unsupervised training-based algorithms need very large sets of data for training but also for validation purpose. High quality methodology requires standardized multi-parametric imaging acquisition protocols and solid diagnostic methods including multiple reader assessment, follow-up imaging, and/or anatomopathological. Multi-center AI studies and pooled imaging data could be an effective solution to spare time and financial resources.

### Limitations and Strengths

The most significant limitation of this review is a wide diversity from one article to another in terms of textural parameters and methods used, which meant that even for similar subjects, it was challenging to aggregate and compare the articles between them. Secondly, the scale used to assess the quality of the articles was practical but rather simplistic. This score made it possible to evaluate many articles with high reproducibility at the expense of a thorough analysis of the methods. At the same time, to the best of the authors’ knowledge, this is the first systematic review in the scientific literature focusing on the use of AI in radiological HCC detection and characterization, omitting pathology and prognosis. This allowed for a detailed analysis that described all the scientific techniques and efforts studied in this narrow field, providing an overview that can provide points for reflection and guide future research.

## 5. Conclusions

Our systematic approach has shown that previous works in HCC detection and characterization have assessed the comparability of conventional interpretation with machine learning using US, CT, and MRI. The distribution of the imaging techniques in our analysis reflects the usefulness and evolution of medical imaging for the diagnosis of HCC. Moreover, our results highlight an imminent need for data sharing in collaborative data repositories to minimize unnecessary repetition and wastage of resources.

## Figures and Tables

**Table 1:** Breakdown of Articles by Year of Publication.

Levels	Counts	% of Total	Cumulative %
1998	1	2.2%	2.2%
2003	1	2.2%	4.3%
2006	2	4.3%	8.7%
2008	1	2.2%	10.9%
2010	1	2.2%	13.0%
2011	1	2.2%	15.2%
2012	1	2.2%	17.4%
2013	1	2.2%	19.6%
2014	2	4.3%	23.9%
2015	1	2.2%	26.1%
2017	3	6.5%	32.6%
2018	4	8.7%	41.3%
2019	10	21.7%	63.0%
2020	7	15.2%	78.3%
2021	5	10.9%	89.1%
2022	5	10.9%	100.0%
Median	2019		

**Table 2:** “Number of Images” Score.

Levels	Counts	% of Total	Cumulative %
0	4	8.7%	8.7%
1	6	13.0%	21.7%
2	36	78.3%	100.0%
Mean: 1.70	Median: 2.00	SD: 0.628	

**Table 3:** “Cohort for Validation” Score.

Levels	Counts	% of Total	Cumulative %
0	20	43.5%	43.5%
1	24	52.2%	95.7%
2	2	4.3%	100.0%
Mean: 0.609	Median: 1.00	SD: 0.577	

**Table 4:** “Year of Publication” Score.

Levels	Counts	% of Total	Cumulative %
1	6	13.0%	13.0%
2	40	87.0%	100.0%
Mean: 1.87	Median: 2.00	SD: 0.341	

**Table 5:** Total Score.

Levels	Counts	% of Total	Cumulative %
1	1	2.2%	2.2%
2	2	4.3%	6.5%
3	7	15.2%	21.7%
4	16	34.8%	56.5%
5	18	39.1%	95.7%
6	2	4.3%	100.0%
Mean: 4.17	Median: 4.00	SD: 1.04	

**Table 6:** Contingency Tables; Total Score and Year.

	Year		χ^2^ Tests		Year		χ^2^ Tests		Year		χ^2^ Tests		Year		χ^2^ Tests		Year		χ^2^ Tests	
Total Score	1998	2003	2006	2008	2010	2011	2012	2013	2014	2015	2017	2018	2019	2020	2021	2022	Total	Value	df	*p*
1	1	0	0	0	0	0	0	0	0	0	0	0	0	0	0	0	1	111	75	0.004
2	0	0	0	0	0	0	0	0	1	0	0	0	1	0	0	0	2			
3	0	0	1	1	1	0	0	0	0	1	2	0	1	0	0	0	7			
4	0	1	1	0	0	0	1	0	0	0	1	3	3	2	1	3	16			
5	0	0	0	0	0	1	0	1	1	0	0	1	5	3	4	2	18			
6	0	0	0	0	0	0	0	0	0	0	0	0	0	2	0	0	2			
Total	1	1	2	1	1	1	1	1	2	1	3	4	10	7	5	5	46			

## Supplementary Materials

The following supporting information can be downloaded at: https://www.mdpi.com/article/10.3390/jcm11216368/s1, File S1: Search Strategy.
