# Supplementary material for: Artificial Intelligence in the Diagnosis of Hepatocellular Carcinoma: A Systematic Review
Source: J Clin Med. 2022 Oct 28;11(21):6368. doi: 10.3390/jcm11216368 (PMC9655417; doi:10.3390/jcm11216368)
Supplement: Supplementary file 1 [file jcm-11-06368-s001.zip › jcm-1940528-supplementary/jcm-1940528-supplementary-File S1.pdf]

Thursday - 5 / May / 2022

((Artificial Intelligence) OR (Machine Learning)) AND ((Hepatocellular Carcinomas) OR (HCC) OR (Liver Cancer))

PubMed
("artificial intelligence"[MeSH Terms] OR ("artificial"[All Fields] AND "intelligence"[All Fields]) OR "artificial intelligence"[All Fields] OR ("machine learning"[MeSH Terms] OR ("machine"[All Fields] AND "learning"[All Fields]) OR "machine learning"[All Fields])) AND ("carcinoma, hepatocellular"[MeSH Terms] OR ("carcinoma"[All Fields] AND "hepatocellular"[All Fields]) OR "hepatocellular carcinoma"[All Fields] OR ("hepatocellular"[All Fields] AND "carcinomas"[All Fields]) OR "hepatocellular carcinomas"[All Fields] OR "HCC"[All Fields] OR ("liver neoplasms"[MeSH Terms] OR ("liver"[All Fields] AND "neoplasms"[All Fields]) OR "liver neoplasms"[All Fields] OR ("liver"[All Fields] AND "cancer"[All Fields]) OR "liver cancer"[All Fields]))
Results: 1,677

Scopus
(TITLE-ABS-KEY (artificial AND intelligence) OR TITLE-ABS-KEY (machine AND learning) AND TITLE-ABS-KEY (hepatocellular AND carcinomas) OR TITLE-ABS-KEY (hcc) OR TITLE-ABS-KEY (liver AND cancer))
Results: 1,426

Cochrane
((Artificial Intelligence) OR (Machine Learning)) AND ((Hepatocellular Carcinomas) OR (HCC) OR (Liver Cancer))
Results: 57 (12 reviews; 1 protocol; 44 trials)

Total: 3,160
Duplicates: 1,052
After removing duplicates: 2,108
